# Use of FibroScan-AST (FAST) Score and Fibrosis-4 Index to Identify Advanced Liver Fibrosis in Patients with Type 2 Diabetes and Metabolic Dysfunction-Associated Steatotic Liver Disease

**DOI:** 10.3390/jcm15010050

**Published:** 2025-12-21

**Authors:** Abir Alsaid, Reem J. Al Argan, Yasir A. Elamin, Nora Alshiekh, Amna Hassan, Abdullah Alotaibi, Ihab Gaarour, Mona H. Ismail

**Affiliations:** 1 Division of Pulmonary, Department of Internal Medicine, King Fahd Hospital of the University, Al Khobar 31952, Saudi Arabia; ahsaid@iau.edu.sa; 2 College of Medicine, Imam Abdulrahman Bin Faisal University, Dammam 31441, Saudi Arabia; rjalarqan@iau.edu.sa (R.J.A.A.); yaelamin@iau.edu.sa (Y.A.E.); adotaibi@iau.edu.sa (A.A.); 3 Division of Endocrine, Department of Internal Medicine, King Fahd Hospital of the University, Al Khobar 31952, Saudi Arabia; 4 Division of Gastroenterology, Department of Internal Medicine, King Fahd Hospital of the University, Al Khobar 31952, Saudi Arabia; noorahaad2018@gmail.com (N.A.); aohassan@iau.edu.sa (A.H.); 5 Department of Anesthesia, King Fahd Hospital of the University, Al Khobar 31952, Saudi Arabia; drg3ror@yahoo.com

**Keywords:** FibroScan-AST score, advanced fibrosis, type 2 diabetes, metabolic dysfunction–associated steatotic liver disease, noninvasive liver tests, Saudi Arabia

## Abstract

**Background:** Metabolic dysfunction-associated steatotic liver disease (MASLD) is highly prevalent in patients with type 2 diabetes (T2D), and advanced fibrosis is the strongest predictor of liver-related morbidity and mortality. Therefore, early noninvasive risk stratification is critical. While the Fibrosis-4 (FIB-4) index and vibration-controlled transient elastography (VCTE) are widely used, the newer FibroScan-AST (FAST) score has shown promise in detecting at-risk metabolic-associated steatohepatitis (MASH) with significant fibrosis. Evidence comparing the FAST and FIB-4 indices in Middle Eastern T2D populations remains limited. We compared the diagnostic performances of these models for advanced fibrosis in Saudi patients with T2D and MASLD. **Methods:** We conducted a retrospective analysis of 273 patients diagnosed with T2D and MASLD. All patients underwent VCTE. To identify advanced fibrosis, we used liver stiffness measurement (LSM) as a surrogate marker for liver biopsy. We calculated the FAST and FIB-4 indices for each patient. To assess the diagnostic performance of these scores, we evaluated their sensitivity, specificity, positive predictive value, negative predictive value, and area under the receiver operating characteristic curve (AUROC). **Results:** In this cohort study, 26.4% of participants had a high-risk FAST score (>0.35; median: 0.13). Patients with high-risk FAST scores (>0.35) were younger, had higher BMIs, elevated liver enzyme levels, and poorer glycemic control than those in the lower-risk groups. High-risk FAST scores were strongly correlated with elevated LSM, FIB-4, and controlled attenuation parameter values (*p* < 0.001). The FAST score demonstrated better performance than the FIB-4 index in detecting advanced fibrosis. It showed higher accuracy (85.4% vs. 77.3%), sensitivity (82.0% vs. 48.0%), and negative predictive value (95.5% vs. 87.8%) while maintaining a similar specificity. The AUROC values were 0.936 (95% CI: 0.901–0.971) for the FAST score compared to 0.711 (95% CI: 0.625–0.797) for the FIB-4 index. **Conclusions:** The FAST score demonstrated better diagnostic accuracy than the FIB-4 index and identified patients with poor metabolic control and obesity as being at the highest risk among Saudi patients with T2D and MASLD. These findings support the integration of other elastography-based tests into stepwise fibrosis screening pathways for diabetic populations, potentially improving the early detection of advanced fibrosis and patient outcomes.

## 1. Introduction

Metabolic dysfunction-associated steatotic liver disease (MASLD) is increasingly recognized as a global health concern, particularly in individuals with type 2 diabetes (T2D) [1]. MASLD affects up to 70% of individuals with T2D, reflecting the strong interplay between insulin resistance, hepatic steatosis, and metabolic dysfunction [2,3,4]. In Saudi Arabia, where T2D affects 16.4% of adults [5] and the incidence of obesity is estimated to be 20–39% [6], the burden of MASLD is substantial. Projections suggest that, by 2030, the number of MASLD cases could increase to approximately 12.5 million, with metabolic dysfunction-associated steatohepatitis (MASH) cases expected to nearly double, accompanied by a sharp increase in advanced fibrosis, cirrhosis, and liver-related mortality [7]. These trends underscore the urgent need for effective strategies to identify patients at the greatest risk of fibrosis progression.

Advanced fibrosis is the strongest predictor of both liver-related and extrahepatic outcomes, including cardiovascular events, malignancy, and all-cause mortality [8,9]. Despite liver biopsy being the reference standard for fibrosis staging, its limitations, including invasiveness and sampling variability, have prompted the development of accurate, reproducible, and noninvasive tests (NITs) for the detection of advanced fibrosis in clinical practice. Consequently, current clinical algorithms emphasize noninvasive, stepwise approaches, especially in high-risk groups, such as individuals with T2D. These algorithms typically begin with simple serum-based indices, most commonly the fibrosis-4 (FIB-4) index, followed by elastography in patients with indeterminate or high-risk results [10]. International guidelines from the American Diabetes Association, the American Association for the Study of Liver Diseases, and the European Association for the Study of the Liver recommend the FIB-4 index as a first-line screening tool for at-risk groups, including those with T2D, given its high negative predictive value (~93%) for excluding advanced fibrosis (≥F3) [11,12,13]. Recently, the FibroScan-AST (FAST) score was introduced to identify patients with active steatohepatitis and significant fibrosis (NAFLD activity score (NAS) ≥ 4, ≥F2), demonstrating a robust diagnostic performance across multiple cohorts [14]. Collectively, these tools reflect the growing potential of NIT-based pathways for incorporating fibrosis risk stratification into diabetes care.

However, the performance of noninvasive tests in patients with T2D remains poor. Diabetes-related factors, including older age, higher body mass index (BMI), systemic inflammation, and multiple comorbidities, can affect serum marker levels and liver stiffness measurements, leading to reduced specificity in patients with diabetes compared with non-diabetic populations. For example, the discriminatory ability of FIB-4-based pathways is significantly lower in T2D patients (c-statistic, 0.68 vs. 0.85) [15]. These limitations underscore the need for more tailored diagnostic approaches in patients with T2D. The development of diabetes-specific cutoffs or the incorporation of additional biomarkers could potentially improve the performance of noninvasive tests in patients with T2D. Furthermore, the combination of multiple noninvasive tests may offer a more comprehensive assessment of liver fibrosis in this population.

To date, few studies have systematically evaluated the diagnostic performance of the FIB-4 index and FAST score in patients with T2D and MASLD, and data from Saudi Arabia or the broader Middle East region, with some of the highest global rates of obesity and diabetes, are lacking. Addressing this gap is essential, as region-specific evidence is needed to guide clinical implementation and optimize case-finding strategies in the future. Therefore, this study aimed to evaluate and compare the performance of the FAST score and FIB-4 index in detecting advanced fibrosis in patients with T2D and MASLD.

## 2. Materials and Methods

### 2.1. Study Design

This retrospective cohort study included all adults (≥18 years) with T2D who attended the Hepatology Clinic at King Fahad Hospital of the University between 1 January 2021 and 31 July 2023. Patients were excluded if they had a history or evidence of viral hepatitis B or C, alcohol consumption, secondary causes of hepatic steatosis, or the use of steatogenic medications (such as amiodarone, tamoxifen, methotrexate, or steroids). Additional exclusion criteria were pregnancy, other hepatic diseases, hepatocellular carcinoma, decompensated cirrhosis, and advanced cardiac disease.

Demographic variables (sex, age, and nationality), anthropometric measurements (body mass index [BMI] and weight), medical history (including systemic arterial hypertension, dyslipidemia, cardiovascular disease, and end-stage renal disease [ESRD]), and diabetes treatment modalities (insulin use, oral hypoglycemic agents, or dietary management) were collected systematically. Laboratory parameters were extracted from the hospital’s information system. These included hemoglobin, platelet count, total bilirubin, serum albumin, alanine aminotransferase [ALT], aspartate aminotransferase [AST], gamma-glutamyl transferase [GGT], international normalized ratio [INR], total cholesterol, low-density lipoprotein [LDL], high-density lipoprotein [HDL], triglyceride [TG], serum creatinine, glycated hemoglobin [HbA1c], and fasting glucose levels. ALT and AST were used as markers of hepatocellular activity. ALT was classified as normal or elevated according to our laboratory reference ranges and sex-specific thresholds (ALT > 30 U/L in men and >19 U/L in women). Hepatic steatosis was initially screened using ultrasonography to detect increased liver echogenicity.

### 2.2. Characteristics of the Study Participants and Procedures

Fibrosis and steatosis were assessed using vibration-controlled transient elastography (VCTE) with controlled attenuation parameter (CAP) technology. Liver stiffness measurement (LSM) and CAP determination were performed by a single, experienced operator (M. I.) using a FibroScan 502 Touch device (Echosens, Paris, France) equipped with both M and XL probes. The M probe was initially used, and the XL probe was automatically selected by the device’s probe selection tool when needed, such as in cases of obesity or large waist circumference. VCTE was considered reliable if ≥10 valid measurements, a success rate of ≥60%, and an interquartile range (IQR) divided by the median (IQR/median) of ≤0.30 were obtained. The LSM staging thresholds for fibrosis staging were defined as follows: F0–F1, <8.2 kPa; F2, 8.2–9.6 kPa; F3, ≥9.7 kPa; and F4, ≥13.6 kPa [16]. MASLD was defined as a CAP score ≥ 274 dB/m, in accordance with validated thresholds [17]. CAP grades were recorded as S1 ≥ 274 dB/m, S2 ≥ 290 dB/m, and S3 ≥ 302 dB/m.

### 2.3. FAST Score Risk Stratification

The FAST score was calculated using LSM (kPa), CAP (dB/m), and AST (U/L) as described by Newsome et al. [14]. AST values were collected ≤30 days after the VCTE examination. FAST score categories were low (≤0.35), indeterminate (0.35–0.67), and high (≥0.67) risk, indicating a risk of MASH with significant fibrosis (stage F2) (nonalcoholic fatty liver disease (NAFLD) activity score (NAS) ≥ 4, F ≥ 2).

### 2.4. FIB-4 Index Risk Stratification

The FIB-4 index was calculated using the formula: (age × AST)/(platelets × √ALT) [18] and further adjusted for age thresholds following McPherson et al. [19]. Patients were stratified into risk groups for advanced fibrosis (≥F3) using age-adjusted thresholds: for <65 years, low < 1.3, indeterminate 1.3–2.67, and high > 2.67; for ≥65 years, low < 2.0, indeterminate 2.0–2.67, and high > 2.67. Laboratory tests were performed ≤ 30 days after the VCTE examination.

### 2.5. Comparison of FAST Scores and the FIB-4 Index

Both indices were evaluated using the same LSM-defined reference standard.

- F ≥ 2: LSM ≥ 8.2 kPa
- F ≥ 3: LSM ≥ 9.7 kPa

Because the FAST score incorporates LSM, we prespecified a potential incorporation bias and treated the FIB-4 index as an unbiased comparator. Secondary analyses included combined strategies (e.g., using the FIB-4 index as a first-line triage, applying the FAST score to indeterminate/high FIB-4 index results, or “both-positive” rules for high specificity).

### 2.6. Data Analysis

Categorical variables are summarized as frequencies and percentages, whereas continuous variables are expressed as means ± standard deviations (SDs) or medians with interquartile ranges (IQRs), depending on their distribution. Group comparisons for categorical variables were performed using the chi-square or Fisher’s exact test, and continuous variables were compared using the Student’s *t*-test or Mann–Whitney U test, as appropriate. In the absence of liver biopsy data, VCTE-derived LSM values were used as a noninvasive surrogate for histologic fibrosis severity. We used a dichotomous threshold for the FAST score, with ≤0.35 indicating rule-out (low risk) and >0.35 indicating rule-in or high risk (intermediate and high risk combined). The diagnostic performance of the FAST score and FIB-4 index for detecting advanced fibrosis was evaluated by calculating the sensitivity, specificity, positive predictive value (PPV), and negative predictive value (NPV), each reported with 95% confidence intervals (CIs). Receiver operating characteristic (ROC) curves were constructed, and the area under the ROC curve (AUROC) was calculated to assess diagnostic performance. Correlations between the FAST score, LSM, and FIB-4 index were assessed. All analyses were conducted using SPSS version 27.0 (IBM Corp., Armonk, NY, USA), and statistical significance was set at *p* < 0.05.

#### Ethics Approval

This retrospective study was approved by the Institutional Review Board of Imam Abdulrahman bin Faisal University (IRB No. IRB-2023-01-319) dated 16 August 2023. Given the retrospective nature of the study and the use of de-identified data, written informed consent was waived. The study was conducted in accordance with the principles of the Declaration of Helsinki.

## 3. Results

### 3.1. Baseline Patient Characteristics

A total of 273 patients met our inclusion criteria. The mean age was 52.9 ± 12.8 years; the majority were female (54.9%), Saudi nationals (86.1%), obese (64.8%), and had an HbA1c level of ≥7 (59.6%). The mean BMI was 34.1 ± 7.5, and 64.8% of the patients were categorized as obese (Table 1). The main comorbidities were hypertension (52.7%), dyslipidemia (45.8%), and history of CVD (17.8%). The most frequent diabetes treatment was oral hypoglycemic medication (58.2%), followed by insulin (35.5%) and a diabetic diet only (18.7%) (Table 1).

**Figure 1:**
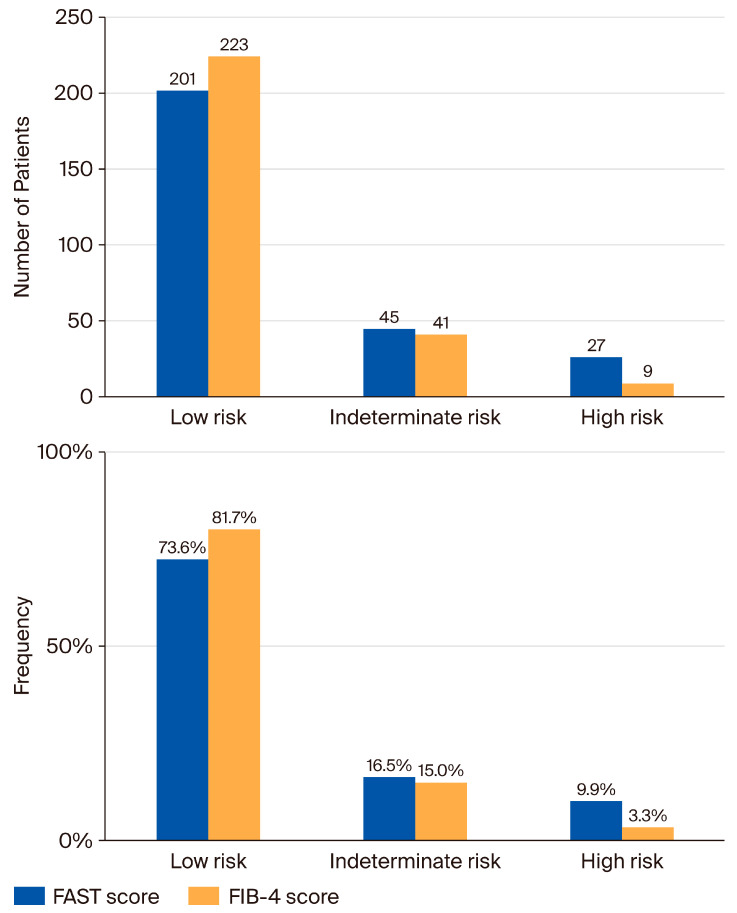
Comparison of the FAST score and FIB-4 index in classifying patients according to liver fibrosis status. FAST, FibroScan-AST; FIB-4, fibrosis 4.

Risk stratification, clinical characteristics, and laboratory assessment based on the FAST score and FIB-4 index: The median FAST score in the total cohort was 0.13 (IQR: 0.05–0.38). Of these, 201 patients (73.6%) were classified as low-risk (FAST ≤ 0.35), 45 (16.5%) as indeterminate (FAST 0.35–0.67), and 27 (9.9%) as high-risk (FAST ≥ 0.67) (Figure 1). For secondary binary analyses, we considered a FAST score > 0.35 as a “rule-in” factor for high risk; 72 patients (26.4%) required further evaluation, comprising 27 in the high-risk category and 45 in the indeterminate-risk category.

Patients with a high-risk FAST score were younger, with a mean age of 51.1 (±12.8) years, and were mostly male (30.9%), Saudi nationals (28.1%), and obese (29.4%). Patients with high-risk FAST scores had a higher BMI and weight (BMI: 36.0 ± 9.3 vs. 33.4 ± 6.7, *p* = 0.010; weight: 97.5 ± 27.9 kg vs. 87.8 ± 18.3 kg, *p* = 0.001, respectively), used less insulin (34.0% vs. 66.0%, *p* = 0.033), and were less likely to be on a diabetic diet only (7.8% vs. 92.2%, *p* = 0.001) compared to those with a low-risk FAST score (Table 1).

Laboratory assessment revealed that patients with high-risk FAST scores had significantly higher HbA1c ≥ 7 (*p* < 0.001) and fasting blood glucose 147 mg/dL (*p* = 0.002) levels, lower platelet counts (245.8 ± 93.4 × 10^9^/µL; *p* = 0.020), and higher transaminase levels (median ALT 63 IU/L, AST 42 IU/L, GGT 75 IU/L; all *p* < 0.001). Serum albumin levels were lower (*p* = 0.045), whereas hemoglobin levels were slightly higher (*p* = 0.042) in the high-risk group than in the low-risk group (Table 2).

### 3.2. Comparison Between the FAST Score, LSM, and FIB-4 Index

The FAST score, LSM by transient elastography (TE), and FIB-4 index showed significant differences between patients with high- and low-risk FAST scores. The median FAST score was 0.59 (0.48–0.75) in the high-risk FAST score group and 0.08 (0.04–0.15) in the low-risk FAST score group (*p* < 0.001). The median LSM was 11.2 (7.2–18.8) kPa. Similarly, the median CAP was 313 dB/m (277–352), and 63.7% of patients had moderate-to-severe steatosis (CAP > 290 dB/m). The median FIB4 index was 0.81 (IQR: 0.57–1.23), and 60 patients (22.0%) exceeded the ≥1.3 threshold, indicating a high risk of advanced fibrosis. When stratified by FAST score risk, the high-risk group had significantly higher LSM, FIB-4, and CAP values than the low-risk group (*p* < 0.001 for all comparisons) (Table 3).

Diagnostic accuracy of the FAST score and FIB-4 index.

**Figure 2:**
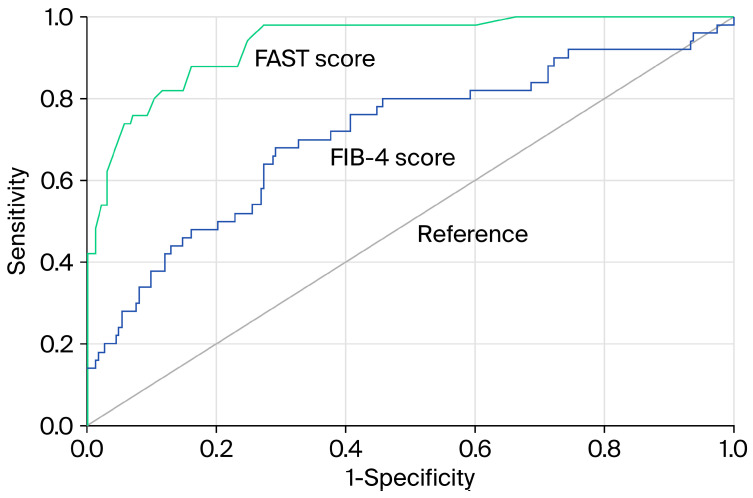
Diagnostic performance of the FAST score versus the FIB-4 index. Receiver operating characteristic (ROC) curves comparing the FAST score (green line) and FIB-4 index (blue line) for the detection of advanced fibrosis in patients with type 2 diabetes and MASLD (*n* = 273). FAST, FibroScan-AST; FIB-4, fibrosis 4.

**Figure 3:**
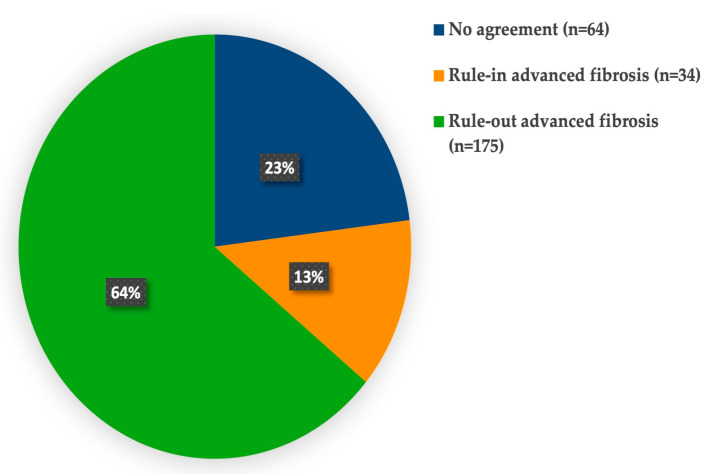
Agreement between the FAST score and FIB-4 index. Both tests classified 64% of the patients as low risk and 13% as high risk, with discordant results observed in 23% of patients. Abbreviations: FAST, FibroScan-AST; FIB-4, fibrosis 4.

At the predefined threshold, the FIB-4 index had a sensitivity of 48.0%, specificity of 83.9%, PPV of 40.0%, and NPV of 87.8% (Table 4). The FIB-4 index demonstrated modest diagnostic performance, with an AUROC of 0.711 (95% CI: 0.625–0.797) (Table 5). In comparison, the FAST score achieved a greater discriminatory ability, with an AUROC of 0.936 (95% CI: 0.901–0.971) (Figure 2), a sensitivity of 82.0%, specificity of 86.1%, PPV of 56.9%, and NPV of 95.5% (Table 4 and Table 5). As expected, the FAST score showed a stronger correlation with LSM than the FIB-4 index (*r* = 0.67 vs. 0.23), and both correlations were statistically significant (*p* < 0.001). Agreement analysis revealed that 77% of patients were classified similarly by both tests: 64% were ruled out, and 13% were ruled in for advanced fibrosis (Figure 3). However, because the FAST score includes LSM as a component, these results may be inflated by incorporation bias and should be interpreted with caution.

## 4. Discussion

This study provides real-world evidence supporting the diagnostic utility of the FAST score for identifying advanced fibrosis in patients with type 2 diabetes and MASLD. In this high-risk cohort, FAST demonstrated superior diagnostic performance compared with the FIB-4 index, with higher AUROC, greater sensitivity, and stronger negative predictive value while maintaining comparable specificity. These findings suggest that FAST may be more effective than FIB-4 for risk stratification in diabetic populations, in whom noninvasive fibrosis assessment remains clinically challenging.

Our results are consistent with prior studies demonstrating the limitations of FIB-4 in populations with metabolic dysfunction and the superior performance of FAST in identifying advanced fibrosis. In this cohort, the FAST score achieved higher AUROC (0.936 vs. 0.711), greater sensitivity (82.0% vs. 48.0%), and stronger NPV (95.5% vs. 87.8%), while maintaining comparable specificity. In a biopsy-confirmed cohort of 279 patients with MASLD, FAST outperformed FIB-4 in terms of AUROC and sensitivity, with similar specificity [20]. Similarly, biopsy-based studies by Castera et al. reported AUROC values of approximately 0.81 for FAST, with consistently higher sensitivity than FIB-4 in patients with T2D [21,22]. Woreta et al. further demonstrated that FAST outperformed FIB-4 in a biopsy-confirmed MASLD cohort [23]. Collectively, these data support the growing evidence that FIB-4 underperforms in high-risk metabolic populations owing to its lower sensitivity and discriminatory capacity. Furthermore, genetic predispositions and specific metabolic profiles in Saudi patients with T2D may explain why the FAST performs better in this group than in others, and therefore warrant validation in prospective studies.

Our findings are further supported by population-based data from Canivet et al. [24], who evaluated multistep screening strategies for MASLD using the U.S. NHANES cohort. In that study, FAST demonstrated higher diagnostic performance than FIB-4 for identifying high-risk MASH, although sensitivity was lower than that observed in our cohort. This discrepancy likely reflects differences in disease prevalence and metabolic risk burden. Notably, both studies relied on transient elastography (LSM ≥ 8 kPa) and composite noninvasive scores rather than liver biopsy, reflecting real-world clinical practice while limiting histological validation.

Patients with elevated FAST scores in our cohort exhibited a distinct metabolic phenotype characterized by younger age, higher BMI, elevated transaminases, and poorer glycemic control. Tada et al. [25] identified BMI, diabetes status, age ≥ 50 years, and ALT levels as independent predictors of liver stiffness in a cohort of 1562 patients with MASLD. Alfadda et al. [26] reported that obesity (BMI ≥ 30) and poor glycemic control were strong predictors of steatosis and fibrosis in a Saudi population with T2D, whereas age was not independently associated with fibrosis severity. Together, these observations underscore the importance of targeted reduction in metabolic risk in patients with T2D and MASLD.

The key strength of our study lies in validating the FAST score against the FIB-4 index in Saudi patients with T2D and MASLD, a high-risk and underrepresented population. However, this study had several limitations. First, the retrospective single-center design may introduce selection and referral bias, potentially limiting generalizability. Second, advanced fibrosis was defined using VCTE rather than liver biopsy, which remains the gold standard, although liver stiffness measurement is widely validated and reflects real-world clinical practice. Third, none of our patients received MASLD-modifying therapies, which can influence liver fibrosis and steatosis. Finally, the study population was primarily composed of Saudi patients with T2D and MASLD; therefore, extrapolation of these findings to other ethnicities or clinical settings should be undertaken with caution.

## 5. Conclusions

The FAST score outperformed the FIB-4 index in identifying advanced fibrosis in Saudi patients with T2D and MASLD, a high-risk and underrepresented population. These findings should be interpreted in the context of the study’s retrospective single-center design, the use of LSM as a surrogate for liver biopsy, and the restricted study population. While the results support the clinical utility of FAST as a pragmatic noninvasive risk-stratification tool in this specific setting, prospective multicenter studies with biopsy confirmation and broader ethnic representation are required to confirm generalizability and clinical impact.

## Figures and Tables

**Table 1:** Demographic and clinical characteristics of patients with type 2 diabetes according to FAST score category (*n* = 273).

	Total (N = 273)	High Risk FAST Score (>0.35)	
		Yes (N = 72)	No (N = 201)
Sex			
Male	123 (45.1%)	38 (30.9%)	85 (69.1%)
Female	150 (54.9%)	34 (22.7%)	116 (77.3%)
Age			
Mean ± SD *	52.9 ± 12.8	51.1 ± 12.8	53.6 ± 12.7
Age group			
<50	101 (37.0%)	31 (30.7%)	70 (69.3%)
50–60	93 (34.1%)	20 (21.5%)	73 (78.5%)
>60	79 (28.9%)	21 (26.6%)	58 (73.4%)
Nationality			
Saudi	235 (86.1%)	66 (28.1%)	169 (71.9%)
Non-Saudi	38 (13.9%)	6 (15.8%)	32 (84.2%)
Height (cm)	162.9 ± 10.3	164.3 ± 10.1	162.4 ± 10.3
Weight (kg) *	90.4 ± 21.6	97.5 ± 27.9	87.8 ± 18.3
Body mass index (kg/m^2^) *	34.1 ± 7.5	36.0 ± 9.3	33.4 ± 6.7
Obesity			
No	96 (35.2%)	20 (20.8%)	76 (79.2%)
Yes	177 (64.8%)	52 (29.4%)	125 (70.6%)
Comorbidity			
Number of diseases	2.2 ± 1.0	2.2 ± 1.0	2.2 ± 1.0
Hypertension	144 (52.7%)	37 (25.7%)	107 (74.3%)
Dyslipidemia	125 (45.8%)	30 (24.0%)	95 (76.0%)
History of CVD	48 (17.8%)	15 (31.3%)	33 (68.8%)
ESRD	19 (7.0%)	4 (21.1%)	15 (78.9%)
Diabetes treatment			
Insulin use *	97 (35.5%)	33 (34.0%)	64 (66.0%)
OHA use	159 (58.2%)	44 (27.7%)	115 (72.3%)
Diabetic diet only *	51 (18.7%)	4 (7.8%)	47 (92.2%)

FAST, FibroScan-AST; CVD, cardiovascular disease; ESRD, end-stage renal disease; OHA, oral hypoglycemic agent. * indicates *p*-value was statistically significant.

**Table 2:** Laboratory measures among patients with type 2 diabetes according to the FAST score category (*n* = 273).

	Total (N = 273)	High-Risk FAST Score (>0.35)	
		Yes (N = 72)	No (N = 201)
Hemoglobin (g/dL) *			
Mean ± SD	13.2 ± 1.9	13.6 ± 1.9	13.0 ± 1.9
Platelets (k/mL) *			
Mean ± SD	266.1 ± 86.5	245.8 ± 93.4	273.4 ± 82.9
Total bilirubin (mg/dL)			
Median (IQR)	0.6 ± 0.4	0.6 ± 0.4	0.5 ± 0.4
Albumin (g/dL) *			
Median (IQR)	3.9 (3.6–4.2)	3.8 (3.4–4.1)	3.9 (3.6–4.2)
AST (U/L) *			
Median (IQR)	21 (17–30)	42 (31–56)	19 (15–23)
ALT (U/L) *			
Median (IQR)	31 (21–48)	63 (42–85)	25 (18–35)
GGT (U/L) *			
Median (IQR)	38 (25–68)	75 (46–193)	31 (23–49)
INR			
Median (IQR)	1.0 (0.9–1.0)	1.0 (1.0–1.0)	1.0 (0.9–1.0)
Total cholesterol (mg/dL)			
Median (IQR)	172 (146–200)	166 (142–197)	173 (149–201)
LDL (mg/dL)			
Median (IQR)	106 (81–128)	98 (74–126)	108 (83–129)
HDL (mg/dL)			
Median (IQR)	45 (37–54)	42 (35–52)	45 (38–55)
Triglycerides (mg/dL)			
Median (IQR)	122 (83–165)	123 (89–182)	121 (83–163)
Creatinine (mg/dL)			
Median (IQR)	0.80 (0.68–1.00)	0.83 (0.67–1.00)	0.79 (0.68–1.02)
HbA1c *			
Median (IQR)	7.2 (6.4–8.7)	8.0 (7.1–9.8)	7.0 (6.3–8.1)
Fasting blood glucose (mg/dL) *			
Median (IQR)	124 (103–171)	147 (113–195)	119 (102–160)

SD, standard deviation; IQR, interquartile range; FAST, FibroScan-AST; AST, aspartate aminotransferase; ALT, alanine aminotransferase; GGT, gamma-glutamyl transferase; INR, international normalized ratio; LDL, low-density lipoprotein; HDL, high-density lipoprotein; HbA1c, glycated hemoglobin. * indicates *p*-value was statistically significant.

**Table 3:** Liver fibrosis measures in patients with type 2 diabetes according to the FAST score category.

	Total (N = 273)	High Risk FAST Score (>0.35)		*p* Value
		Yes (N = 72)	No (N = 201)	
FAST, median (IQR)	0.13 (0.05–0.38)	0.59 (0.48–0.75)	0.08 (0.04–0.15)	<0.001
LSM (kPa)				
Median (IQR)	5.7 (4.3–7.8)	11.2 (7.2–18.8)	5.1 (4.1–6.1)	<0.001
F1 No or mild fibrosis (<8.2)	212 (77.7%)	26 (12.3%)	186 (87.7%)	<0.001
F2 Significant fibrosis (8.2–9.6)	11 (4.0%)	5 (45.5%)	6 (54.5%)	
F3 Advanced fibrosis (9.7–13.5)	20 (7.3%)	14 (70.0%)	6 (30.0%)	
F4 Cirrhosis (≥13.6)	30 (11.0%)	27 (90.0%)	3 (10.0%)	
LSM (kPa)				
F1–2 No advanced fibrosis (<9.7)	223 (81.7%)	31 (13.9%)	192 (86.1%)	<0.001
F3–4 Advanced fibrosis (≥9.7)	50 (18.3%)	41 (82.0%)	9 (18.0%)	
FIB-4				
Median (IQR)	0.81 (0.57–1.23)	1.26 (0.82–1.61)	0.71 (0.51–0.96)	<0.001
Low risk (<1.3/2.0)	223 (81.7%)	40 (17.9%)	183 (82.1%)	<0.001
Indeterminate risk (≥1.3/2.0)	41 (15.0%)	25 (61.0%)	16 (39.0%)	
High risk (≥2.67)	9 (3.3%)	7 (77.8%)	2 (22.2%)	
FIB-4				
Low risk (<1.3)	213 (78.0%)	38 (17.8%)	175 (82.2%)	<0.001
High risk (≥1.3)	60 (22.0%)	34 (56.7%)	26 (43.3%)	
CAP (dB/m)				
Median (IQR)	313 (277–352)	350 (310–382)	297 (269–339)	<0.001
S0 No steatosis (<274)	64 (23.4%)	9 (14.1%)	55 (85.9%)	<0.001
S1 Mild steatosis (274–290)	35 (12.8%)	2 (5.7%)	33 (94.3%)	
S2 Moderate steatosis (291–302)	21 (7.7%)	4 (19.0%)	17 (81.0%)	
S3 Severe steatosis (>302)	153 (56.0%)	57 (37.3%)	96 (62.7%)	
CAP (dB/m)				
S0–1 No or mild steatosis (≤290)	99 (36.3%)	11 (11.1%)	88 (88.9%)	<0.001
S2–3 Moderate or severe steatosis (>290)	174 (63.7%)	61 (35.1%)	113 (64.9%)	
FibroScan Probe				
M	138 (50.5%)	34 (24.6%)	104 (75.4%)	0.510
XL	135 (49.5%)	38 (28.1%)	97 (71.9%)	

FIB-4, fibrosis 4; LSM, liver stiffness measurement; CAP, controlled attenuation parameter

**Table 4:** Comparison of the accuracy of the FAST score and FIB-4 index in detecting liver fibrosis (*n* = 273).

	FAST Score	FIB-4 Index	*p* Value
Diagnostic accuracy	85.4%	77.3%	<0.001
Sensitivity	82.0%	48.0%	<0.001
Specificity	86.1%	83.9%	0.508
Positive predictive value	56.9%	40.0%	0.053
Negative predictive value	95.5%	87.8%	0.005

**Table 5:** Area under the curve for predicting advanced fibrosis (*n* = 273).

	Area Under the Curve	95% Confidence Interval		*p* Value
		Lower Limit	Upper Limit	
FAST score	0.936	0.901	0.971	<0.001
FIB-4	0.711	0.625	0.797	<0.001

Abbreviations: FAST, FibroScan-AST; FIB-4, fibrosis 4.

## Data Availability

The datasets generated and analyzed during the current study are not publicly available due to limitations imposed by our hospital’s privacy policies. However, data are available from the corresponding author (mismail.md@gmail.com) upon obtaining appropriate Institutional Review Board approval at Imam Abdulrahman bin Faisal University.
